# Influence of Normal Aging and Multisensory Data Fusion on Cybersickness and Postural Adaptation in Immersive Virtual Reality

**DOI:** 10.3390/s23239414

**Published:** 2023-11-26

**Authors:** Marie-Philippine Séba, Pauline Maillot, Sylvain Hanneton, Gilles Dietrich

**Affiliations:** Institut des Sciences du Sport Santé de Paris (URP 3625), Université Paris Cité, F-75015 Paris, France; pauline.maillot@u-paris.fr (P.M.); sylvain.hanneton@u-paris.fr (S.H.); gilles.dietrich@u-paris.fr (G.D.)

**Keywords:** Immersive Virtual Reality, age, cybersickness, postural control

## Abstract

Immersive Virtual Reality (VR) systems are expanding as sensorimotor readaptation tools for older adults. However, this purpose may be challenged by cybersickness occurrences possibly caused by sensory conflicts. This study aims to analyze the effects of aging and multisensory data fusion processes in the brain on cybersickness and the adaptation of postural responses when exposed to immersive VR. Methods: We repeatedly exposed 75 participants, aged 21 to 86, to immersive VR while recording the trajectory of their Center of Pressure (CoP). Participants rated their cybersickness after the first and fifth exposure. Results: The repeated exposures increased cybersickness and allowed for a decrease in postural responses from the second repetition, i.e., increased stability. We did not find any significant correlation between biological age and cybersickness scores. On the contrary, even if some postural responses are age-dependent, a significant postural adaptation occurred independently of age. The CoP trajectory length in the anteroposterior axis and mean velocity were the postural parameters the most affected by age and repetition. Conclusions: This study suggests that cybersickness and postural adaptation to immersive VR are not age-dependent and that cybersickness is unrelated to a deficit in postural adaptation or age. Age does not seem to influence the properties of multisensory data fusion.

## 1. Introduction

Multisensory data fusion is a process involved in postural stability. Postural stability is ensured via the brain’s continuous integration of several sensory channels. In some cases, the sensory channels’ data are contradictory, provoking significant physiological reactions leading to discomfort and what is called cybersickness. Immersive VR systems are intended to become health tools for motor rehabilitation [1]. Their field of use is gradually expanding towards older adults [2]. Indeed, several studies used Head-Mounted Displays (HMDs) to modify visual inputs and challenge the sensorimotor system in older adults. VR with HMDs can also help to study or assess balance disorders in older adults [3]. For example, the Balance Reeducation Unit, a VR system, can assess and treat balance disorders in older adults with vestibular dysfunctions [4]. A few studies have already demonstrated the benefit of using these systems to prevent falls and reduce the fear of falling in older adults [5].

However, immersive VR systems, especially HMDs, have deleterious side effects, reported mainly in adults. Cybersickness and balance disorders can hinder the use of VR [6,7]. Indeed, cybersickness (nausea, pallor, sweating…) is the leading cause of discontinuation [8]. Cybersickness can be linked to multisensory data fusion characteristics via the Reason and Brand [9] sensory mismatch theory, developed initially for motion sickness. Then, Bos et al. [10] extended this theory to visually induced motion sickness, including cybersickness. Motion sickness susceptibility is an individual predictor of cybersickness [11]. In this theory, cybersickness originates from a mismatch between predicted sensory signals and feedback. Sensory mismatches are perceived via the different sensory modalities causing sensory conflicts, as expressed through the sensorimotor outcomes, which can result in balance disorders [12]. Several human, technological, and environmental factors can mitigate the occurrence and intensity of cybersickness [13,14,15,16]. Human factors relate to individual characteristics [17], such as motion sickness susceptibility [11], gender [18,19], age [20], or gaming experience [18]. Technological factors relate to the immersive VR system specificities, such as the visual field provided by the visual interface [8] or the motion-to-photon latency [21]. Environmental factors relate to the characteristics of the task and the Virtual Environment (VE) design, such as parameters of the visual flow, the gaming content, the level of interaction, the proposed way of displacement, or the length of exposure [8,11,22]. French and Canadian guidelines have been published to limit the onset and severity of cybersickness [23,24].

In addition to cybersickness, VR causes changes in postural stability. Changes in postural parameters while using an HMD indicate that postural stability is modified. However, when wearing an HMD and simply turning it on and off (blank image), the postural parameters are not significantly changed [25]. Somewhat, balance is influenced by the stimulation, especially when there are vections [12,26] and also by the content type: an abstract scene induces more body swaying than a realistic environment [27]. For example, a higher velocity of visual stimulation can increase the body postural response magnitude [28].

Cybersickness and balance disruptions are sensitive to repetition. With repetition, individuals can adapt or at least habituate to sensory conflicts. To our knowledge, habituation to cybersickness with age has not been extensively investigated. However, healthy adult participants treated with galvanic vestibular stimulation while being exposed to a virtual ship’s motion (HMD) needed more sessions than those without the stimulation to habituate to cybersickness (3 days, 15 min/day). Without the stimulation, habituation to cybersickness occurred at the second session, on the 2nd day [29]. Gavgani et al. [30] showed that participants exposed for 15 min during 3 consecutive days to a roller-coaster ride with an HMD could extend their ride tolerance and experience fewer subjective symptoms after exposure. Otherwise, research on other types of visually induced motion sickness showed a peak increase in sickness around 15 min and a decrease after 20 min of exposure [31,32,33]. Individuals are more likely to adapt their postural stability faster than for cybersickness. When exposed to a roller coaster VE with an HMD, the first exposure induces postural disruptions that can be damped down after 4 to 5 exposures [12].

Repeating exposures can lead to cybersickness habituation and adaptation to postural disruptions in healthy adults. However, with aging and sensorimotor aging, adaptation and habituation capabilities can be altered [34,35]. As a result, the responses to visual disturbances caused by VR HMD may be more intense with aging. Sensory conflicts take longer to process with advancing age and sensory reliability is modified [36]. The degeneration of somatokinesthetic modalities occurs and increases the importance of visual signals for postural stability [37]. Advancing age modifies sensory recalibration efficiency, which is essential for controlling posture. There is also a slower downweighing of inconsistent visual signals. On the other hand, Wiesmeier et al. [38] found that proprioceptive information in older adults is up-weighted compared to visual or vestibular information. They also measured a higher reaction time, compromising the stability of feedback systems in older adults. All of these age-related modifications in the sensorimotor system could accentuate the VR side effects in older adults. Modifications in the sensorimotor and balance control systems, such as the slowing down and modified multisensory integration with advancing age, could be linked to the effects observed between cybersickness, balance disruptions, and aging. 

In older adults, VR systems have already been used to identify aging-related sensorimotor changes. Balance disturbances were recorded in older adults using HMDs [3]. A VR study demonstrated that older adults use more visual feedback than younger adults to control their balance. Postural stability increases with supplementary visual feedback [39]. Evidence regarding the expression of cybersickness with VR-HMD in older adults are still scarce. Although motion sickness decreases with age [40], a few recent studies available on cybersickness and age do not reach a global consensus [22,41,42]. For example, when exposed to mainstream virtual environments with an HMD, older adults (M = 71.3) expressed less cybersickness than their younger counterparts (M = 19.7) [43]. Cybersickness is less expressed in subjects over 35 than in subjects under 35 when exposed to scenic or gaming content with an HMD [22]. However, when controlling navigation while in VR-HMD, participants aged over 30 are more likely to suffer from cybersickness [11]. Exposing older adults to a loss of balance and to cybersickness symptoms could impact their willingness to use HMDs. If aging does not allow for adaptation or at least habituation to the provoked sensory mismatches, this technology could not be recommended for older adults. 

Therefore, our study investigates the capabilities to adapt to sensory conflicts caused by HMDs with healthy aging. Adaptation is investigated by analyzing individual postural stability and cybersickness symptoms induced by recurrent exposures with an HMD. In this study, we repeatedly exposed participants to a passive VR scenario using an HMD to investigate (i) whether cybersickness is age-related, (ii) whether there is an age-related postural adaptation following repeated exposures, and (iii) whether postural adaptation results in a decrease in cybersickness.

## 2. Materials and Methods

### 2.1. Participants

The procedure was reviewed by the Ethics Committee Sud Méditerranée IV (ID-RCB 2019-A01843-54, 2 November 2020) and explained to the participants who signed a consent form. Participants had to be in good health to be included in the study. They were included if they ranked their general health status on 4 ‘good’ or 5 ‘very good’ on a Likert scale ranging from 1 ‘very bad’ to 5 ‘very good’ [44]. Participants with balance-related disorders could not enter the study, including visual or vestibular impairments (except participants with corrected-to-normal vision) or any condition that would prevent participants from standing during an extended period. Participants who did not complete the experiment were excluded. The data were obtained from 75 healthy subjects (male: 29; female: 46) aged 21 to 86 (M = 42.04 ± 17.82). Our sample of participants can be divided into three age categories for information purposes only. Twenty-eight participants aged 21 to 29 (M = 23.93 ± 2.16) can be identified as young. Twenty participants aged 30 to 49 (M = 40.50 ± 6.20) can be identified as middle-aged. Twenty-seven participants aged 50 and older (M = 61.96 ± 10.15) can be identified as older adults. The older adult category’s lower limit is based on the assumption that postural control performance decreases at age 50 [45].

### 2.2. Experimental Procedure

**Figure 1:**
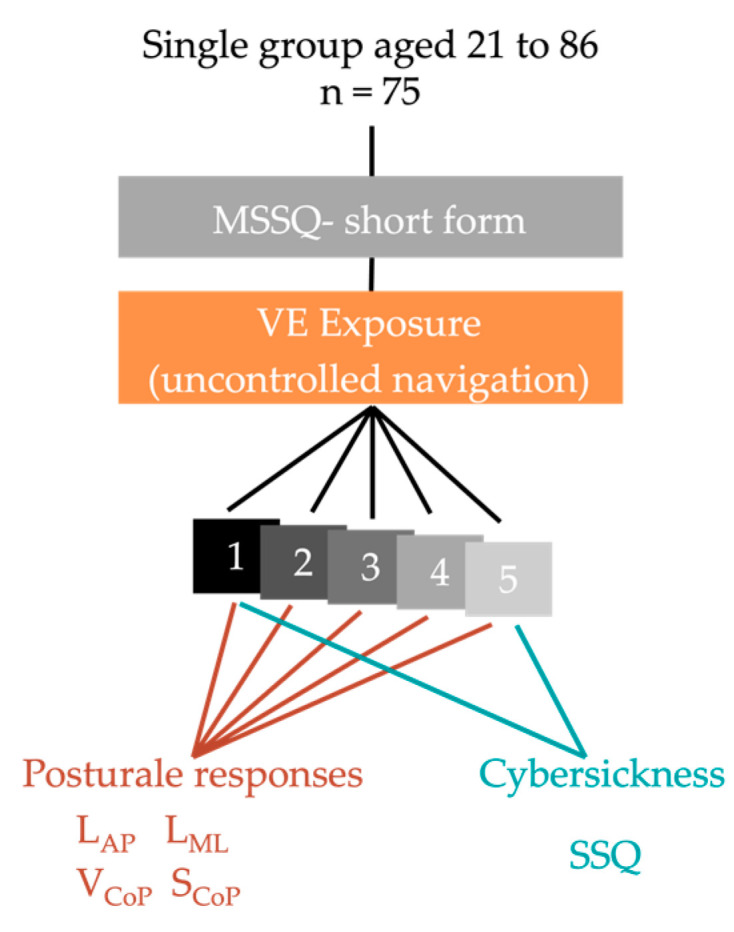
Schematic of the experimental protocol. Note. MSSQ: motion sickness susceptibility questionnaire; VE: virtual environment; 1: exposure number; SSQ: simulator sickness questionnaire; L_AP_: anteroposterior center of pressure trajectory length; L_ML_: mediolateral center of pressure trajectory length; V_CoP_: center of pressure mean velocity; S_CoP_: 95% confidence ellipse surface.

The main goal of this empirical research was to analyze cybersickness and postural changes during repeated exposures to a VE (Figure 1). Participants underwent the same VR exposure five times. Each VR exposure lasted 4 min and 10 s. The accumulated time of these five exposures was 20 min and 50 s. Participants could rest between the five VR exposures and remove the HMD.

#### 2.2.1. Virtual Reality System

**Figure 2:**
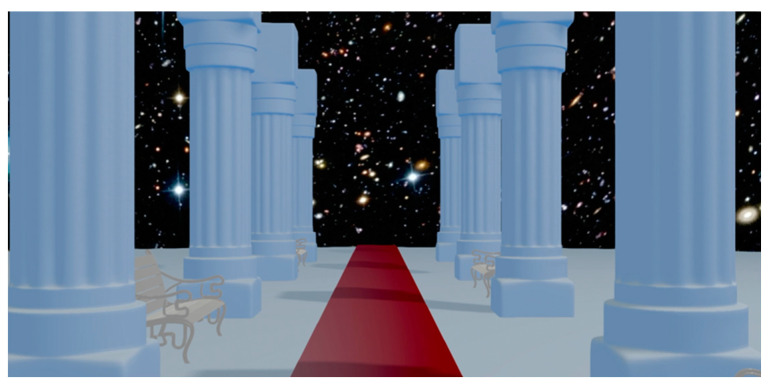
A picture of the virtual environment as seen by the participant.

Regarding the VR setup, the HMD used to broadcast the VE was an Oculus Rift (Meta©, consumer version 1, released in 2016, Irvine, CA, USA). The HMD had a resolution of 1080 × 1200 pixels per eye, a 110° field of view, a refresh rate of 90 Hz, and a 6-degrees-of-freedom movement sensitivity. The HMD was connected to a computer ASUS G11CD-K-FR047T Intel Core i7-7700, Windows 10 (Asus©, Taipei, Taiwan) with an NVIDIA graphic card GeForce GTX 1070 (Samsung©, Suwon, Republic of Korea, and TSMC©, Hsinchu, Taiwan). Blender© (Blender Online Community, Amsterdam, The Netherlands) and Blend4Web© (Triumph LLC©, Moscow, Russia) were used to build and render the VE. The VE consisted of alternating benches and columns on either side of a rectangular red carpet (Figure 2). A night sky with stars surrounded the environment. Participants were immersed in uncontrolled navigation in a first-person perspective and could change the camera viewpoint orientation by moving their heads in the yaw, pitch, and roll axis. The first-person viewpoint was programmed to produce movements that induced vections (forward, backward, rotations, up, and down). Therefore, it was a situation of passive exposure.

#### 2.2.2. Cybersickness Assessment 

Participants rated their cybersickness after the first (SSQ1) and fifth (SSQ2) exposure using a French version [46] of Kennedy et al. [47]’s Simulator Sickness Questionnaire (SSQ). The SSQ is a 16-item questionnaire covering symptoms of cybersickness. Participants rated the severity of their symptoms from ‘not at all’ to ‘severely.’ We applied the method suggested by Kennedy et al. [47] to calculate the total cybersickness scores. The categorization of subjective symptoms is as follows: 0 = no symptoms, <5: negligible symptoms, 5–10: minimal symptoms, 10–15: significant symptoms, 15–20: symptoms are a concern, and >20: a problem simulator. We assessed the amplitude of habituation to cybersickness by computing a cybersickness habituation score, defined as the subtraction of SSQ2 minus SSQ1. Since motion sickness susceptibility is an individual predictor of cybersickness, we also assessed participants’ motion sickness susceptibility. The French version of the Motion Sickness Susceptibility Questionnaire-short form (MSSQ) assessed motion sickness susceptibility using the MSSQ raw score [46,48]. The MSSQ raw score is a summation of motion sickness susceptibility experienced as a child (MSA-Child) and experienced over the last ten years (MSB-Adult).

#### 2.2.3. Balance Assessment 

During each trial, the CoP was recorded using a force platform (Wii Balance Board, Nintendo, Kyoto, Japan). This force platform is considered to be a reliable tool for assessing quiet standing balance [49]. Recordings were made at a re-sampled rate of 60 Hz. Participants were standing barefoot on the force platform with no foot placement constraint and had to remain stationary. The CoP trajectory length on the anteroposterior (L_AP_) and mediolateral (L_ML_) axis, the mean velocity of the CoP (V_CoP_), and the 95% confidence ellipse surface of the CoP (S_CoP_) were the postural parameters used to measure postural stability (Prieto et al., 1996). Moreover, we introduced a postural adaptation score to assess the amplitude of adaptation. This postural adaptation score is the subtraction of the values of the fifth exposure, minus the values of the first exposure for each postural parameter.

## 3. Results

Statistical analyses were performed using JASP software (Version 0.16.2) [50]. The statistical figures were extracted from this software. Since several studies pointed out that the SSQ data do not follow a normal distribution [51,52], we assessed the distribution of our variables. The SSQ data, as well as the postural measures, do not follow a normal distribution. Then, only non-parametric statistical tests were performed.

### 3.1. Cybersickness with Repetition

First, a Friedman test performed between SSQ1 (M = 20.65 ± 19.95) and SSQ2 (M = 30.02 ± 30.55) shows a significant increase in cybersickness between the first and fifth exposure (χ^2^ = 9.00, *p* = 0.003, W = 0.12). Cybersickness significantly increases with five repeated exposures.

### 3.2. Cybersickness, Motion Sickness, Habituation Score, and Age

We searched for Spearman correlations between SSQ1 and age and between SSQ2 and age. The correlations were not significant (respectively, ρ = −0.11, *p* = 0.35; ρ = −0.18, *p* = 0.12). Cybersickness does not correlate significantly with age in our study. Moreover, we searched for Spearman correlations between the habituation score and age. The habituation score does not significantly correlate with age (ρ = −0.12, *p* = 0.31). Age has no influence on cybersickness habituation within five repeated exposures. The MSSQ raw score (M = 11.73 ± 9.98) was not correlated with participants’ age (ρ = −0.13, *p* = 0.28). The MSA-Child (ρ = −0.14, *p* = 0.24) and MSB-Adult (ρ = −0.11, *p* = 0.33) scores were also uncorrelated with age.

### 3.3. Postural Reference Measures

Then, we computed four reference indexes, R-L_ML_, R-L_AP_, R-V_CoP_, and R-S_CoP_, to assert the differences between the postural reference measures. The indexes were the mean of the considered variable over the five exposures without the imposed vections, with the participant standing still. The environment without imposed vections consisted of the first 30 s of the trial. Spearman’s rho correlations were performed between the reference indexes and age. R-L_AP_ and R-V_CoP_ are significantly correlated with age (respectively, ρ = 0.28, *p* = 0.014; ρ = 0.23, *p* = 0.049). Age influences postural stability when exposed to a virtual environment without vections. 

### 3.4. Postural Changes with Repetition

**Figure 3:**
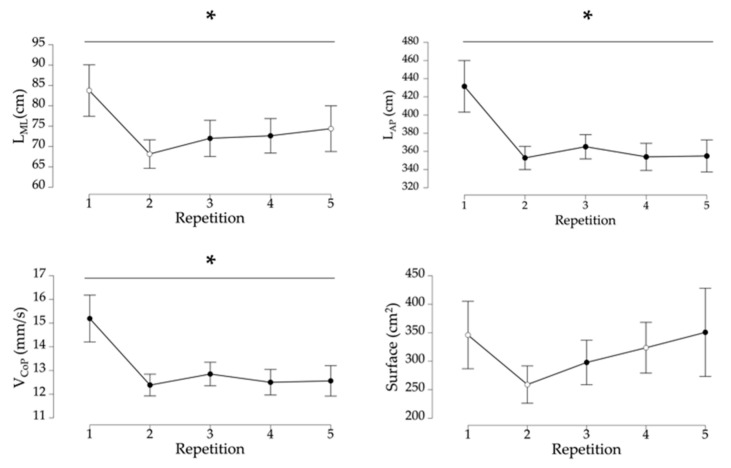
Graphs of the significant effect of repetition shown for L_ML_, L_AP_, and V_CoP_ (from left to right) and correlations with age. Note. *, significant effect of repetition; ○, non-significantly correlated with age; ●, significantly correlated with age. L_ML_: mediolateral center of pressure trajectory length; L_AP_: anteroposterior center of pressure trajectory length; V_CoP_: center of pressure mean velocity.

A Friedman test assessed the effect of repetition on the different postural parameters. Repetition has a significant effect on L_ML_, L_AP_, and V_CoP_ (respectively, χ^2^ = 21.97, *p* < 0.001, W = 0.07; χ^2^ = 31.78, *p* < 0.001, W = 0.11; and χ^2^ = 30.56, *p* < 0.001, W = 0.10). Repetition tends to decrease the parameters. The effect size for these parameters is medium. However, the effect of repetition is not significant for S_CoP_ (χ^2^ = 8.16, *p* = 0.09) (Figure 3). Postural stability increases with repetition when exposed to vections.

### 3.5. Postural Changes and Age

We performed Spearman correlation tests between age and postural parameters for each repetition (Figure 3). Age is significantly correlated with L_AP_ and V_CoP_ for all repetitions (*p* < 0.05). Age is significantly correlated with L_ML_ for the third (ρ = 0.24, *p* < 0.05) and fourth repetition (ρ = 0.25, *p* < 0.05). Age is significantly correlated with S_CoP_ for the third (ρ = 0.29, *p* = 0.013) and fifth repetition (ρ = 0.23, *p* = 0.043). Age influences postural stability when exposed to vections. 

### 3.6. Postural Adaptation Score and Age

The postural adaptation score was calculated for L_ML_, L_AP_, V_CoP_, and S_CoP_ by subtracting the values obtained at the fifth repetition from those obtained at the first repetition. This postural adaptation score was then correlated with age. None of the correlations were significant. The amplitude of postural adaptation does not depend on age in this study.

### 3.7. Evolution of Postural Adaptation and Cybersickness Habituation Scores

Spearman correlations were performed between the postural adaptation scores for L_ML_, L_AP_, V_CoP_, and S_CoP_ and the habituation score. None of the correlations were significant. No evidence was found concerning relationships between the evolution of cybersickness and the evolution of postural parameters. Postural adaptation and cybersickness habituation are not related within five repeated exposures. 

### 3.8. Predicting Cybersickness from Postural Parameters

Participants with SSQ scores above 15 were classified as sick (‘symptoms are a concern’), and those below 15 were classified as non-sick. State 1 refers to SSQ1 and state 2 refers to SSQ2. We performed a logistic regression to assess whether the postural outcomes, L_ML_ and L_AP_, at the first and fifth exposure can predict participants who get sick after the first and fifth exposures. Logistic regressions were performed to ascertain the effects of postural control on the likelihood of developing cybersickness after the first and fifth exposure. The best models were obtained to predict state 2. The logistic regression model to predict state 1 with L_ML_ in the first exposure was nonsignificant (χ^2^(73) = 0.35, Nagelkerke R^2^ = 0.006, *p* = 0.56). The logistic regression model to predict state 1 with L_AP_ in the first exposure was nonsignificant (χ^2^(73) = 0.12, Nagelkerke R^2^ = 0.002, *p* = 0.73). However, the logistic regression model for state 2 with L_ML_ in the fifth exposure was significant (χ^2^(73) = 7.69, Nagelkerke R^2^ = 0.13, *p* = 0.006). The L_ML_ coefficient was significant (weight z = 2.29; *p* = 0.022). The model correctly classified 44 of the 46 sick participants. However, it failed to classify 6 out of 29 non-sick participants. For L_AP_, the logistic regression model for state 2 in the fifth exposure was significant as well (χ^2^(73) = 10.41, Nagelkerke R^2^ = 0.18, *p* = 0.001). The L_AP_ coefficient was significant (weight z = 2.90; *p* = 0.004). The model correctly classified 40 of the 46 sick participants. Nevertheless, it also failed to classify the non-sick participants, 11 out of 29. Interestingly, the probability of being sick decreases with L_AP_. According to the results, the postural outcomes at the first exposure cannot predict participants’ sickness state after a single exposure. The probability of sickness seems to significantly depend on the L_ML_ and the L_AP_ postural parameters at the fifth exposure. However, the poor predictive power of the models for non-sick participants suggests that other factors intervene.

### 3.9. Cybersickness and Motion Sickness Susceptibility

SSQ1 (ρ = 0.29, *p* = 0.049) and SSQ2 (ρ = 0.35, *p* < 0.001) are significantly correlated with the motion sickness susceptibility raw score, but correlation coefficients are low. We also performed a logistic regression to ascertain the effects of motion sickness susceptibility on the likelihood of developing cybersickness after the first and fifth exposure. The same classification was used to separate participants according to whether they were sick. The logistic regression model for state 1 with MSSQ was nonsignificant (χ^2^(73) = 2.13, Nagelkerke R^2^ = 0.037, *p* = 0.14). The model was significant only for state 2 with the MSSQ score as the input (χ^2^(73) = 11.35, Nagelkerke R^2^ = 0.191, *p* < 0.001). The MSSQ coefficient was significant (weight z = −2.89; *p* = 0.004). The model correctly classified 37 of the 46 sick participants and 17 non-sick participants out of 29. Motion sickness susceptibility is not a strong enough predictor for cybersickness. 

## 4. Discussion

Our study investigated the influence of age on multisensory data fusion processes and the capability to adapt to immersive VR. Adaptation was investigated by analyzing the cybersickness and postural parameters changes induced by repeated exposures with an HMD. 

The proposed VE induced cybersickness in most of the participants. After the first exposure, 35 participants out of 75 had a SSQ score greater than 15, while there were 46 after the fifth exposure. The means of SSQ1 (M = 20.65 ± 19.95) and SSQ2 (M = 30.02 ± 30.55) are respectively greater than 20 and 30. According to the categorization of symptoms proposed by the authors [47], the SSQ scores correspond to the categorization ‘A problem simulator.’ As a reminder, the first exposure only lasted 4 min and 10 s. The total exposure time was 20 min and 50 s. Contrary to what was expected according to the literature, the symptoms were already high after the first exposure (SSQ1 > 20; total exposure time < 5 min) and even higher after 20 min (SSQ2 > 30; total time > 20 min) [31]. This high occurrence of cybersickness can be explained by the high vection periods in the trials. In addition, repeated exposures, even with rest times, significantly increased cybersickness among participants. This finding is consistent with other studies where an increase in cybersickness with exposure time is reported [8,53]. Moreover, rests inferior to 2 h between exposures are not enough for cybersickness symptoms to disappear completely, nor enough for habituation to occur. Our results do not favor habituation to cybersickness when repeating the exposure on the same day and resting less than one hour between exposures. Habituation usually occurs within days [29]. 

To further investigate the relationship between cybersickness habituation and age, we introduced a cybersickness habituation score, which was shown not to be significantly related to age. The results of this study do not support a higher expression of cybersickness with age. The VE proposed in this experiment, with short translations and rotation movements, induced cybersickness enough to occur in most of the participants despite the state of their sensorimotor system (SSQ1 > 20: 36/75; SSQ2 > 30: 46/75). Thus, aging of the sensorimotor system does not appear to worsen nor predict the expression of cybersickness symptoms in this experiment. 

Regarding postural changes, repeating the exposure allowed for an adaptation to the postural responses induced by the VE. A single exposure allowed participants to decrease their L_ML_, L_AP_, and V_CoP_ significantly at the second exposure. Fransson et al. [12] demonstrate a postural adaptation with repetition. Although a decrease in postural parameters is not always linked to a good balance, it can be interpreted as an adaptation process when exposed to a new stimulus. An efficient postural control could be defined by one’s capacity to react and to set up a relevant dynamic vis-à-vis a conflicting stimulus between visual, proprioceptive, and vestibular sensory cues. In this study, we found this kind of adaptation since L_AP_ and V_CoP_ returned close to their reference indexes starting at the second exposure.

Then, several postural parameters are modulated by age. Regarding the four reference indexes, the control measure was recorded while participants were wearing the HMD with the scene turned on, without imposed vections. We chose this reference measure to include the postural changes induced by simply wearing the HMD [25]. Only R-L_AP_ and R-V_CoP_ correlated with age. This result is in accordance with the observed increase in postural parameters with age, especially when the visual inputs are disturbed [54]. However, this control measure needs to be interpreted cautiously since the VE scene displayed during the control measure was non-photorealistic and abstract VEs can induce more postural disruptions than photorealistic ones [27]. Regarding the imposed vections, L_AP_ and V_CoP_ are significantly correlated with age during each exposure. This means that the length and velocity of the CoP increase significantly with the age of participants. The weakness of correlation coefficients might be explained by the fact that postural control is impaired differently and variably in individuals, depending on the decade, starting as early as the fifth decade [55]. However, according to the literature, postural adaptation capabilities remain similar throughout the lifespan [56]. To investigate changes in postural adaptation capabilities, we defined a postural adaptation score and used it to assess amplitudes of adaptation. Indeed, this postural adaptation score was not significantly correlated with age. 

To further investigate whether cybersickness is related to postural parameters, we used a logistic regression to predict the probability of being sick. However, our regression model revealed significant only for L_ML_ and L_AP_ for state 2, after the fifth exposure. The more L_ML_ and L_AP_ are low, the greater the probability of being sick. This result echoes the ‘VR-lock’ strategy [26], where sick participants would increase their postural stability to manage cybersickness. 

Other individual characteristics could influence cybersickness and postural changes, such as field dependency [57], the cognitive influence of movement belief [58], or the brain’s ability to reweigh sensory information, especially vestibular sensitivity. Subjective vection sensitivity [53] can be influenced by aging and could influence adaptation and habituation capabilities. Moreover, in our study, we found that there was a correlation between MSSQ and SSQ1 and SSQ2. Motion sickness susceptibility can help predict cybersickness after 20 min of repeated exposures, but this individual characteristic is not enough to predict cybersickness accurately. This relationship between motion sickness susceptibility and cybersickness has already been reported [11]. These results may suggest that cybersickness and motion sickness share a common sensorimotor pathway. Due to the absence of a significant correlation with age, age is not interfering with it.

Finally, several postural parameters were found to be significantly related to age during trials, whereas cybersickness was not. Visual disturbances challenge postural stability, especially with age. Although postural disruptions and cybersickness occur, the adaptation and habituation mechanisms do not seem to be on the same level of control. Postural adaptation could be faster than habituation to cybersickness. To confirm this hypothesis, repeated exposures on several days and recordings of postural responses and cybersickness for individuals of different ages would be needed (similarly to [29,30]). Furthermore, electromyography measures of the lower limbs could allow for an investigation of the ‘VR lock’ strategy in response to cybersickness with aging.

Regarding the limitations of our study, a couple can be highlighted. Firstly, although the SSQ is the most commonly used tool in the literature [14], it would have been interesting to specifically assess cybersickness during exposure using a different tool, such as the Fast Motion Sickness Scale [59] and the Cybersickness in Virtual Reality Questionnaire [60]. However, assessing cybersickness during exposure influences postural responses and can potentially introduce a bias. This is why we chose to assess cybersickness after exposure in our study. Secondly, we did not consider fatigue. Increased fatigue may have increased cybersickness, as fatigue is a specific item of the SSQ. However, to limit fatigue, participants were allowed to rest between exposures. Our participants did not feel very fatigued, since they took short 5 min rests on average, except for one participant who had to take an urgent and long phone call. Thirdly, another concern is about sex differences in cybersickness. Females have been identified as more susceptible to cybersickness than males with HMD [19], especially due to their different computing and gaming experiences [18]. There are observed differences between the two genders, but the origins of these differences remain unclear [61,62].

## 5. Conclusions

Although with aging, sensorimotor abilities are degraded, (i) cybersickness is not related to age and there is no significant habituation to cybersickness. Regarding postural responses, postural oscillations in the anteroposterior axis were shown to be more important. This study shows that (ii) an adaptation to the postural disruptions generated via immersive VR is possible, even for older people. Finally, (iii) the postural adaptation observed is not related to a decrease in cybersickness. In our results, age was not found to be related to the amplitude of postural adaptation nor the evolution of cybersickness with repetition. Age does not seem to fundamentally influence the properties of multisensory data fusion via the central nervous system. However, from a clinical point of view, postural disruptions due to VR are likely to be expected in people of any age and need to be supervised for their security, as well as for cybersickness symptoms. 

## Data Availability

Data are available on request from the corresponding author.
